# Use of Cell Saver in Elective Coronary Bypass Surgery: What Do We Risk When Saving Blood?

**DOI:** 10.3390/jcm14124230

**Published:** 2025-06-13

**Authors:** Adem Reyhancan, Mürsel Büyükadalı, Ertuğrul Koçak, Orkut Güçlü, Serhat Hüseyin, Suat Canbaz

**Affiliations:** Department of Cardiovascular Surgery, Faculty of Medicine, Trakya University, 22030 Edirne, Türkiye

**Keywords:** cell saver, blood, drainage, albumin, coronary

## Abstract

**Background/Objectives:** Allogeneic transfusion is a commonly used method to replace blood and blood elements lost during cardiac surgery, but it also has quite undesirable effects. The use of Cell Saver is now almost routinely recommended. The aim of this study is to investigate the clinical and laboratory outcomes of the use of Cell Saver in elective CABG. **Methods**: Patients who had undergone elective CABG between January 2022 and October 2024 were retrospectively analyzed, and 344 patients were included in the study. Patients were divided into two groups: Cell Saver used (CS, *n* = 110) and not used (NCS, *n* = 234). The groups were compared in terms of pre- and postoperative clinical and laboratory outcomes. **Results**: The mean age of the cases included in the study was 63.65 ± 9.05 years (340 patients, range 36–87). The mean amount of drainage in the first 6 h postoperatively was 298.18 ± 155.81 mL in the NCS group and 388.64 ± 173.62 mL in the CS group (*p* < 0.001). In the first 24 h, it was 703.22 ± 320.39 mL in the NCS group and 827.73 ± 344.69 mL in the CS group (*p* = 0.001). Prolonged drainage was more frequent in the CS group (*p* = 0.004) and the length of hospital stay was longer (*p* = 0.014). The postoperative albumin level was lower in the CS group (*p* = 0.003). **Conclusions**: Although the use of Cell Saver reduces the need for allogeneic transfusions, it leads to increased bleeding in the initial period, prolonged drainage, and thus a longer hospital stay. In elective procedures, blood management should be evaluated and optimized using all methods.

## 1. Introduction

Coronary artery disease remains a major cause of morbidity and mortality in both developed and developing countries [1]. Coronary artery bypass grafting (CABG) is one of the most commonly performed procedures in the treatment of coronary artery disease. According to the Society of Thoracic Surgeons report, 65–74% of annual cardiac surgeries are isolated CABGs. Approximately 90% of these operations are performed with cardiopulmonary bypass (CPB) [2]. These procedures can result in significant blood loss due to CPB and the type of surgical techniques used [3]. This often necessitates perioperative and postoperative allogeneic blood transfusions. However, allogeneic blood transfusions are associated with several adverse outcomes, including immunomodulation, increased risk of infection, hemolytic reactions, and higher healthcare costs [4,5,6]. Therefore, intraoperative blood management and autologous blood collection have become increasingly important in recent years [7].

The technique of reinfusing lost blood into the patient was proposed by Blundell in the first quarter of the 19th century based on animal experiments, and Dr. Klebanoff was the first to describe the autotransfusion method during surgery using a device [8,9]. The Cell Saver was developed to collect blood spilled during surgery, process it by centrifugation and filtration, and return the red blood cell-enriched autologous blood to the patient. This method is a safe and effective means of reducing the need for allogeneic transfusions [10]. However, the effects of the use of Cell Savers on postoperative laboratory parameters and clinical outcomes is the subject of ongoing debate. In a systematic review by Lloyd et al. [11], randomized controlled trials comparing cases with and without the use of Cell Savers in many surgical disciplines were examined for their impact on outcomes such as blood transfusion rate, myocardial infarction, deep vein thrombosis, cerebrovascular accident (CVA), infection, and mortality in the postoperative period. In cardiac surgery with or without a pump, the effect of Cell Saver on postoperative outcomes has been classified as low or moderate with certainty. There are also opinions linking the use of Cell Saver to an increased tendency to bleed in the postoperative phase of cardiac surgery, partly due to the removal of blood proteins and platelets during blood preparation [12,13]. There are also studies showing different results regarding the effects on bleeding, transfusion volume, and hemoglobin levels in the early postoperative period [12,14,15].

The aim of this study is to investigate the impact of Cell Saver use on postoperative hematologic and biochemical parameters, transfusion requirements, and clinical outcomes in patients undergoing elective isolated CABG surgery to provide objective data for routine use in clinical practice.

## 2. Materials and Methods

### 2.1. The Study Population

All patients who underwent CABG at our centre between January 2022 and October 2024 were retrospectively examined. Data from a total of 436 patients were extracted from the archive files and the automation system. Patients who underwent combined surgery, emergency surgery, or off-pump surgery were excluded from the study. Patients with previous cardiac surgery, permanent pacemaker implantation, chronic atrial fibrillation, and known hereditary coagulation disorders were also excluded. Data were collected from 344 patients who underwent elective isolated on-pump CABG via median sternotomy and met the inclusion criteria. The Cell Saver device was used consecutively in all cardiac surgical procedures. Our centre is a state university hospital and due to reimbursement issues, the Cell Saver device can no longer be used from the first quarter of 2023. In our study, the patients who underwent surgery when the Cell Saver device and disposable sets were not available at our centre were referred to as the “No Cell Saver” group (NCS, *n* = 234), while the patients who used Cell Saver during surgery were referred to as the “Cell Saver” group (CS, *n* = 110).

**Figure 1:**
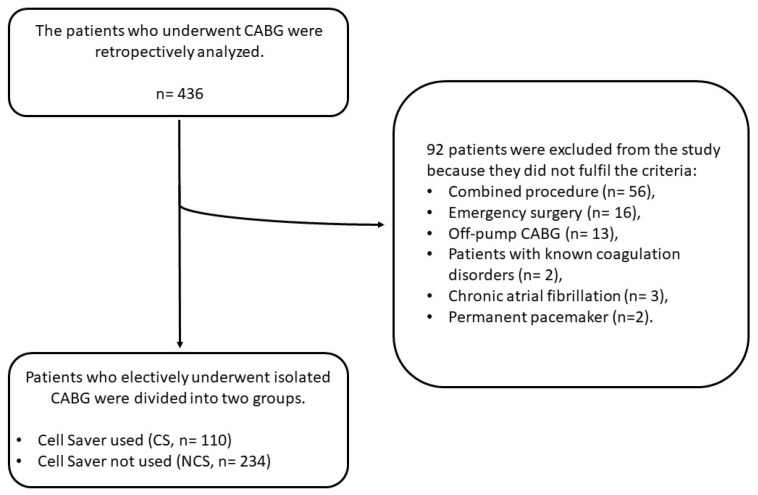
The flowchart for patient selection.

The flowchart for patient selection in the study is shown in Figure 1.

### 2.2. The Use of Cell Saver

All operations were performed via a median sternotomy. In the CS group, blood in the surgical field was aspirated into the Cell Saver device (the Sorin EXTRA^®^ Autotransfusion System (Livanova, London, UK)) from the start of surgery. After systemic heparinization, only the cardiotomy suction was used from the time the activated clotting time (ACT) exceeded 400 s. After completion of the CPB and start of the protamine infusion, the blood in the surgical field was aspirated again and exclusively into the Cell Saver device. The blood remaining in the CPB reservoir was also transferred to the Cell Saver device. The blood collected in the Cell Saver reservoir was processed and reinfused before the patient left the operating theatre. In the NCS group, cardiotomy suction was performed from the time the ACT exceeded 400 s. Cardiotomy suction was continued until the protamine infusion dose had reached half. The blood remaining in the reservoir was administered to the patient via the aortic cannula until the protamine dose was halved, paying attention to systemic arterial pressure and central venous pressure.

### 2.3. Data Collection

Demographic data, preoperative risk evaluations (EuroSCORE II), comorbidities, body surface area (BSA) calculations, ejection fraction, and the largest measured left atrial diameter of the patients were retrospectively analyzed. The number of grafted vessels, CPB, and aortic cross clamping (ACC) time were recorded. ACT values before systemic heparinization, after neutralization with protamine, and during ICU admission were recorded. Drainage volumes at the end of the first 6 and 24 h after surgery were assessed using the intensive care unit (ICU) follow-up charts.

Complete blood count (CBC), coagulation, and other biochemical parameters were determined and recorded for each patient on preoperative day 1 and day 1 and day 4 after surgery. The number of red blood cell (RBC) transfusions received by the patients during their hospitalization was recorded. Transfusions were performed in cases where the hemoglobin level was <8 gr/dL or <9 gr/dL with anemia symptoms such as hypoxia, hypotension, and tachycardia. Postoperative complications such as pneumonia, prolonged drainage, new-onset atrial fibrillation (NOAF), re-exploration, reintubation, acute kidney failure (AKF), CVA, and mortality were recorded during the 30-day postoperative period. Prolonged drainage was defined as the inability to remove the chest tube for more than 4 days due to the amount of drainage. The two groups were compared with regard to these outcomes.

### 2.4. Statistical Analysis

Statistical analysis was performed using SPSS software version 23.0 (IBM Corp., Armonk, NY, USA) for Windows. Descriptive data are presented as mean ± standard deviation (SD) or as number and frequency. The distribution of variables was measured using the Kolmogorov–Smirnov test. The chi-square test was used to analyze independent qualitative data, and Fisher’s exact test was used when the conditions of the chi-square test were not met. Student’s *t*-test was used for independent quantitative data when variances were homogeneously distributed. The Mann–Whitney U-test was used when the variances were not homogeneously distributed. A *p*-value of <0.05 was considered to indicate statistical significance.

## 3. Results

The mean age of the cases included in the study was 63.65 ± 9.05 years (340 patients, range 36–87). There were no significant differences between the groups in terms of demographic data and preoperative risk assessments. The proportion of male patients was higher in the CS group (80.9%) than in the NCS group, but no statistically significant difference was found (*p* < 0.083). The mean ejection fraction and left atrial diameter were, respectively, 54.2 ± 7.4% and 35.9 ± 4.7 mm in the CS group, while they were 53.4 ± 7.8% and 36.7 ± 4.6 mm in the NCS group (*p* > 0.05). When the two groups were compared for the mean number of vessels grafted, no significant difference was found (CS, 3.3 ± 0.8; NCS, 3.2 ± 0.9; *p* > 0.05). CPB and ACC times were similar between the two groups. The demographic data and the comparison of preoperative and intraoperative findings are summarized in Table 1.

In the CS group, the mean amount of blood reinfused into the patients with Cell Saver was 568.25 ± 197.94 (range 160 to 1050 mL). The mean amount of drainage at the end of the first 6 h after surgery was 298.18 ± 155.81 mL in the NCS group and 388.64 ± 173.62 mL in the CS group. At the end of the first 24 h, it was 703.22 ± 320.39 mL in the NCS group and 827.73 ± 344.69 mL in the CS group. Both at the end of the first 6 h and in the first 24 h after surgery, there was statistically significantly more bleeding in the patients in the CS group (*p* < 0.001 for the first 6 h and *p* = 0.001 for the first 24 h). Considering the possible incorrect evaluations arising from the differences in BSA of the patients, the drainage amounts were compared again by proportioning them to BSA, and similarly, the amount of bleeding was found to be higher in the CS group patients. On the other hand, it was found that the mean amount of RBC transfusions in the NCS group was 3.82 ± 2.37 bags, while in the CS group, it was 2.95 ± 2.05 bags (*p* = 0.001). Re-exploration due to bleeding was required in six (2.6%) patients in the NCS group and seven (6.4%) patients in the CS group. Although this occurred more frequently in the CS group in percentage terms, no statistically significant difference was found (*p* > 0.05).

The mean extubation time was 6.14 ± 4.08 h in the NCS group and 6.29 ± 3.87 h in the CS group (*p* > 0.05). When comparing the need for reintubation, no significant difference was found between the two groups. Patients in the NCS group stayed in the ICU for 2.4 ± 2.6 days, while patients in the CS group stayed for 2.2 ± 0.7 days (*p* > 0.05).

Prolonged drainage was observed in 35 (15.2%) patients in the NCS group and in 31 (28.2%) patients in the CS group. Prolonged drainage was statistically significantly more frequent in the CS group (*p* = 0.004). Patients in the NCS group were discharged after 8.5 ± 4.5 days, while this period was 9.9 ± 5.5 days in the CS group. Patients in the CS group had a statistically significantly longer hospital stay (*p* = 0.014).

No significant difference was found between the two groups in terms of pneumonia, NOAF, AKF, CVA, rehospitalization, and mortality in the 30-day postoperative period.

The comparison of clinical outcomes in the postoperative period is shown in Table 2.

ACT levels before systemic heparinization, after protamine neutralization, and on admission to the ICU were compared between groups. The time between the last two ACT values corresponds to the time at which the processed waste blood was transfused to the patients in the CS group. In the CS group, the mean ACT was 124.76 ± 15.28 s before heparinization, 118.92 ± 12.53 s after protamine neutralization, and 122.46 ± 13.53 s after transfusion of the processed blood. In the NCS group, the mean ACT was 126.01 ± 17.84 s before heparinization, 116.79 ± 12.81 s after protaminization, and 117.25 ± 11.99 s on admission to the ICU. A statistically significant increase in ACT was observed after the processed blood transfusion in the CS group (*p* < 0.001).

A mean albumin level of 4.19 ± 0.43 in the NCS group and 4.14 ± 0.45 gr/dl in the CS group was found in the blood samples taken before the operation (*p* > 0.05). On the first postoperative day, it was 3.51 ± 0.28 in the NCS group and 3.41 ± 0.32 gr/dl in the CS group; on the fourth postoperative day, it was 3.29 ± 0.30 in the NCS group and 3.19 ± 0.29 gr/dl in the CS group. On the first and fourth postoperative days, the albumin level was statistically lower in the CS group (*p* = 0.003).

Mean hemoglobin and hematocrit levels were higher in the CS group before surgery than in the NCS group (*p* = 0.039 for hemoglobin; *p* = 0.057 for hematocrit). On the first postoperative day, there was a more significant statistical difference in the mean hemoglobin and hematocrit values of the patients in the CS group (*p* < 0.001 for both). On the fourth postoperative day, no significant difference was found between the two groups in terms of hemoglobin and hematocrit (*p* = 0.316 for hemoglobin; *p* = 0.558 for hematocrit).

While the platelet count was similar between the two groups in the preoperative CBC (*p* >0.05), on the first postoperative day, it was significantly lower in the CS group (*p* = 0.030). On the fourth postoperative day, no statistically significant difference was found between the two groups.

In the blood samples taken before the operation and on the first and fourth postoperative days, no significant difference was found between the groups in terms of CRP and INR values.

The comparison of the two groups in terms of blood test results is summarized in Table 3.

## 4. Discussion

In the present study, the effects of intraoperative autologous blood sampling with a Cell Saver system (CS) were investigated in patients undergoing elective coronary artery bypass grafting (CABG). Our results suggest that although the use of the Cell Saver reduces the need for external blood transfusions, it is associated with increased postoperative bleeding volume, prolonged drainage time, lower platelet and albumin concentrations, and consequently a longer hospital stay.

In particular, the amount of bleeding in the CS group was significantly higher in the early postoperative phase, especially in the first 6 and 24 h. This increase in bleeding was associated with prolonged activated clotting time (ACT) measurements after protamine administration and on admission to the ICU, indicating a delay in clotting. These observations raise concerns about the hemostatic integrity of the Cell Saver-prepared reinfused blood. In addition, platelet counts measured on the first postoperative day were significantly lower in the CS group than in the NCS group, suggesting a dilution or consumption effect possibly due to the washing process. The albumin concentration was also significantly reduced in the CS group on both the first and fourth postoperative days, which may have contributed to the increased bleeding and prolonged recovery times observed in this cohort.

In a randomized controlled study by Scrascia et al. [16], which is similar to our practice, the blood remaining in the CPB reservoir was processed with Cell Saver and returned to a group of patients. In this group of patients, more bleeding was observed in the postoperative period. This was found to be related to increased fibrinolytic activity after the blood processed with the Cell Saver system was returned. In another randomized controlled trial, it was reported that the use of Cell Saver delayed clot formation and reduced the firmness of the clot, resulting in an increase in bleeding in the initial period [17]. Similarly, many studies have concluded that the use of Cell Saver in the initial phase increases bleeding [12,15]. In a prospective observational study, patients operated with the on-pump, off-pump + Cell Saver, and off-pump methods were compared and it was found that the use of Cell Saver induced fibrinolysis as much as CPB [18]. In addition, there are also studies indicating that the use of Cell Saver is not associated with bleeding and actually reduces it [14,19,20]. In our study, the amount of bleeding was significantly higher in the CS group after the first 6 h and at the end of 24 h. A significant difference was observed between ACT after protamine and at the time of ICU admission in the CS group. Although there are studies suggesting that there is no significant amount of residual heparin in the filtered blood of Cell Saver, it was observed in our study that ACT increased after transfusion [21].

Another finding that we observed in our study as a reason for the increased bleeding in the CS group was the decrease in platelet count. On the first day after surgery, the platelet count was lower in the CS group than in the NCS group. Similarly, in the study by Campbell et al. [17], it was observed that the platelet count in the postoperative period was lower in the cases where Cell Saver was used. In another study, more bleeding and lower platelet counts were observed at the end of the first 6 and 24 h in cases where Cell Saver was used [12]. Processing the blood remaining in the CPB reservoir with Cell Saver and transfusing it to the patient also leads to a washout and excretion of platelets in the blood [13].

In the CS group, although the amount of bleeding in the first 24 h after surgery and the frequency of prolonged drainage in the following days were higher, the hemoglobin level in the early phase was higher and the need for allogeneic transfusions was lower. In our study, the hemoglobin level was significantly higher in patients in the CS group in the preoperative phase (*p* = 0.039). However, on the first postoperative day, the statistical difference became more significant (*p* < 0.001). This difference in hemoglobin levels between the two groups was no longer observed on the fourth postoperative day. Similarly to our study, Al-Mandhari et al. [15] reported that bleeding was higher in the cases where Cell Saver was used, but hemoglobin levels were higher in the early phase. In addition, fewer blood transfusions were required in the cases where Cell Saver was used, despite the high bleeding rate. Damgaard et al. [14] found that hemoglobin levels were higher in the cases where Cell Saver was used, although bleeding and blood product requirements were similar. Reyes et al. [12] reported that the bleeding rate was higher in the low-risk cases when Cell Saver was used, but there was no difference between the groups in terms of hemoglobin levels and blood consumption. When these three parameters are taken into account, the exact effect of using Cell Saver is controversial.

Cell Saver prepares an RBC-rich suspension by washing the blood from the surgical field and the CPB reservoir with saline. Pillay et al. [22] showed that the amount of albumin in pump blood washed with saline was significantly reduced. In our study, the amount of albumin in the blood samples taken on the first and fourth postoperative day was significantly lower in the CS group than in the NCS group. Hypoalbuminemia in the postoperative period was associated with increased bleeding in the initial period and prolonged hospital stay [23]. In the CS group, we observed both prolonged drainage and prolonged hospitalization. We think that increased bleeding and hypoalbuminemia in the initial phase led to prolonged drainage and thus prolonged hospitalization.

There are studies indicating that blood transfusions are a risk factor for NOAF regardless of their quantity, while there are also studies indicating that they are not a risk factor for NOAF [24,25,26]. Koçyiğit et al. [27] reported that more blood transfusions were performed in cases with NOAF after CABG. It was also found that less NOAF was observed in cases in which Cell Saver was used than in the cases in which it was not used. There are also studies linking perioperative anemia and allogeneic blood transfusions to an increased incidence of AKF and CVA [28,29]. In our study, no significant difference was found when the CS and NCS groups were compared in terms of the incidence of AKF, CVA, and NOAF in the postoperative period.

### Limitations of This Study

Due to the retrospective design of this study, there is a risk of selection bias in patient selection and data collection. No propensity score matching was applied to adjust for potential confounding variables between groups, which should be considered a factor that may limit internal validity in between-group comparisons. Although the extent of bleeding in the postoperative period was analyzed, advanced coagulation analyzes such as thromboelastography (TEG) or rotational thromboelastometry (ROTEM) were not used for a detailed assessment of hemostatic function, but only routine laboratory parameters. These methodological limitations require a cautious interpretation of the results obtained and limit the possibility to draw causal conclusions.

## 5. Conclusions

In this study, we found that the use of Cell Saver in elective CABG procedures was associated with a reduced need for allogeneic transfusions. However, this was also associated with increased early postoperative hemorrhage, lower albumin levels, and consequently prolonged drainage and hospital stay. One possible factor could be the retransfusion of residual blood in the reservoir after completion of cardiopulmonary bypass with the Cell Saver. As elective CABG procedures are generally associated with relatively low intraoperative blood loss compared to other cardiac surgeries, a comprehensive approach to perioperative blood management—including strategies before, during, and after surgery—may help to optimize outcomes and reduce both transfusion requirements and healthcare costs. Further large-scale, prospective studies are needed to clarify the clinical significance of these findings and to better define the role of Cell Saver in this particular surgical population.

## Figures and Tables

**Table 1:** Preoperative assessment and surgical data.

	NCS (*n* = 234)	CS (*n* = 110)	*p*
Age (year)	63.7 ± 8.9	63.6 ± 9.3	0.966
BSA (m^2^)	1.89 ± 0.17	1.89 ± 0.19	0.830
EuroScore II	1.48 ± 0.95	1.46 ± 0.92	0.849
Biological sex			0.083
Female	65 (27.8%)	21 (19.1%)	
Male	169 (72.2%)	89 (80.9%)	
Comorbidities			
Diabetes mellitus	112 (47.9%)	52 (47.3%)	0.919
Hypertension	168 (71.8%)	80 (72.7%)	0.857
COPD	14 (6%)	8 (7.3%)	0.826
CVA	18 (7.7%)	9 (8.2%)	>0.99
GFR < 50 mL/min	43 (18.4%)	16 (14.5%)	0.468
Ejection fraction (%)	54.2 ± 7.4	53.4 ± 7.8	0.355
LA diameter (mm)	35.9 ± 4.7	36.7 ± 4.6	0.201
Number of grafted vessels	3.2 ± 0.9	3.3 ± 0.8	0.431
CPB time (min)	106.16 ± 30.36	102.88 ± 24.74	0.241
ACC time (min)	54.81 ± 17.38	52.80 ± 14.70	0.294

ACC, aortic cross clamp; BSA, body surface area; COPD, chronic obstructive pulmonary disease; CPB, cardiopulmonary bypass; CVA, cerebrovascular accident; GFR, glomerular filtration rate; LA, left atrium.

**Table 2:** Clinical results in postoperative phase.

	NCS (*n* = 234)	CS (*n* = 110)	*p*
Cell Saver Volume (mL)	0	568.25 ± 197.94	<0.001
Drainage in 6 h (mL)	298.18 ± 155.81	388.64 ± 173.62	<0.001
*Drainage Index (mL/m^2^)*	159.42 ± 85.13	207.23 ± 95.78	<0.001
Drainage in 24 h (mL)	703.22 ± 320.39	827.73 ± 344.69	0.001
*Drainage Index (mL/m^2^)*	374.26 ± 172.89	440.54 ± 192.08	0.002
RBC Transfusion (bag)	3.82 ± 2.37	2.95 ± 2.05	0.001
Extubation Time (h)	6.14 ± 4.08	6.29 ± 3.87	0.750
Reintubation	8 (3.5%)	6 (5.5%)	0.392
Re-exploration	6 (2.6%)	7 (6.4%)	0.126
Prolonged Drainage	35 (15.2%)	31 (28.2%)	0.004
Pneumonia	11 (4.7%)	8 (7.3%)	0.340
NOAF	94 (40.2%)	47 (42.7%)	0.653
AKF	9 (3.9%)	3 (2.8%)	0.759
CVA	3 (1.3%)	0 (0%)	0.554
ICU Stay (day)	2.4 ± 2.6	2.2 ± 0.7	0.470
Hospital Stay (day)	8.5 ± 4.5	9.9 ± 5.5	0.014
Rehospitalization	15 (6.6%)	3 (2.7%)	0.142
Mortality	7 (3%)	0 (0)	0.102

AKF, acute kidney failure; CVA, cerebrovascular accident; ICU, intensive care unit; NOAF, new-onset atrial fibrillation; RBC, red blood cell.

**Table 3:** Comparison of pre- and postoperative blood test results.

	NCS (*n* = 234)	CS (*n* = 110)	*p*
ACT (s)			
*Preoperative*	126.01 ± 17.84	124.76 ± 15.28	0.529
*Post-protamine*	116.79 ± 12.81	118.92 ± 12.53	0.149
*Admission to the ICU*	117.25 ± 11.99	122.46 ± 13.53	<0.001
CRP (mg/L)			
*Preoperative*	16.21 ± 23.87	17.65 ± 26.96	0.616
*Postoperative 1st day*	75.42 ± 30.88	77.70 ± 39.43	0.561
*Postoperative 4th day*	92.38 ± 43.02	96.18 ± 48.93	0.467
Albumin (g/dL)			
*Preoperative*	4.19 ± 0.43	4.14 ± 0.45	0.289
*Postoperative 1st day*	3.51 ± 0.28	3.41 ± 0.32	0.003
*Postoperative 4th day*	3.29 ± 0.30	3.19 ± 0.29	0.003
Hemoglobin (g/dL)			
*Preoperative*	13.12 ± 1.78	13.54 ± 1.73	0.039
*Postoperative 1st day*	9.54 ± 0.69	9.91 ± 0.87	<0.001
*Postoperative 4th day*	10.36 ± 0.99	10.48 ± 0.99	0.316
Hematocrit (%)			
*Preoperative*	38.75 ± 4.99	39.83 ± 4.69	0.057
*Postoperative 1st day*	27.76 ± 2.32	28.96 ± 2.59	<0.001
*Postoperative 4th day*	30.82 ± 3.06	31.03 ± 2.97	0.558
Platelet (10^3^ µL)			
*Preoperative*	279.74 ± 83.04	270.27 ± 76.86	0.313
*Postoperative 1st day*	218.66 ± 64.39	203.45 ± 51.29	0.030
*Postoperative 4th day*	218.59 ± 71.29	207.51 ± 55.21	0.253
INR			
*Preoperative*	1.016 ± 0.076	1.009 ± 0.078	0.453
*Postoperative 1st day*	1.099 ± 0.072	1.099 ± 0.080	0.954
*Postoperative 4th day*	1.049 ± 0.083	1.052 ± 0.111	0.808

ACT, activated clotting time; CRP, C-reactive protein, INR, international normalized ratio.

## Data Availability

The data presented in this study are available on request from the corresponding author.

## Abbreviations

The following abbreviations are used in this manuscript:

ACC	Aortic cross clamping
AKF	Acute kidney failure
ACT	Activated clotting time
CABG	Coronary artery bypass grafting
COPD	Chronic obstructive pulmonary disease
CPB	Cardiopulmonary bypass
CRP	C-reactive protein
CVA	Cerebrovascular accident
GFR	Glomerular filtration rate
INR	International normalized ratio
LA	Left atrium
NOAF	New-onset atrial fibrillation
RBC	Red blood cell
